# Silencing of long non-coding RNA MEG3 alleviates lipopolysaccharide-induced acute lung injury by acting as a molecular sponge of microRNA-7b to modulate NLRP3

**DOI:** 10.18632/aging.103752

**Published:** 2020-08-27

**Authors:** Handi Liao, Suning Zhang, Jianou Qiao

**Affiliations:** 1 Department of Intensive Care Unit, Shanghai Ninth People’s Hospital, Shanghai JiaoTong University School of Medicine, Shanghai 201999, P.R. China; 2 Department of Emergency Medicine, Shanghai Ninth People’s Hospital, Shanghai JiaoTong University School of Medicine, Shanghai 201999, P.R. China; 3 Department of Respiratory Medicine, Shanghai Ninth People’s Hospital, Shanghai JiaoTong University School of Medicine, Shanghai 200011, P.R. China

**Keywords:** long non-coding RNA maternally expressed gene 3, microRNA-7b, NLR pyrin domain containing 3, acute lung injury, lipopolysaccharide

## Abstract

We aimed to elucidate the roles of the long non-coding RNA (lncRNA) maternally expressed gene 3 (MEG3)/microRNA-7b (miR-7b)/NLR pyrin domain containing 3 (NLRP3) axis in lipopolysaccharide (LPS)-induced acute lung injury (ALI). Mouse alveolar macrophage NR8383 and mice were administrated with LPS to establish ALI models *in vitro* and *in vivo*. NLRP3 was silenced while miR-7b was overexpressed in LPS-induced NR8383 cell model of ALI. The interleukin-18 (IL-18) and IL-1β, as well as caspase-1, tumor necrosis factor-α (TNF-α) and IL-6 protein levels were assayed. To further investigate the underlying mechanisms of NLRP3 in ALI, lncRNA MEG3 was silenced and miR-7b was overexpressed in LPS-induced NR8383 cell model of ALI, after which *in vivo* experiments were performed for further verification. NLRP3 was highly expressed in LPS-induced NR8383 cell model of ALI. Silencing NLRP3 or overexpressing miR-7b inhibited IL-18 and IL-1β, as well as caspase-1, TNF-α and IL-6. LncRNA MEG3 could sponge miR-7b, and lncRNA MEG3 silencing or miR-7b overexpression downregulates NLRP3 expression, thus reducing IL-18 and IL-1β, as well as caspase-1, TNF-α and IL-6 levels. The *in vivo* experiments further confirmed the aforementioned findings. Silencing lncRNA MEG3 augments miR-7b binding to NLRP3 and downregulates NLRP3 expression, which ultimately improves LPS-induced ALI.

## INTRODUCTION

Acute lung injury (ALI), characterized by persistent hypoxemia refractory to oxygen supplementation, is a progressive and destructive disease accompanied by typical physiological changes and radiological manifestations [1]. ALI may cause morbidity and mortality among critically ill patients, and lung injury induced by lipopolysaccharide (LPS) is a highly diffused model, which allows for enhanced understanding of the well documented morphological and functional changes associated with ALI [2]. LPS, also known as endotoxin, represents a key component of the outer membrane of gram-negative bacteria. LPS may be implicated in gram-negative bacterial infection processes with studies highlighting their ability to induce inflammation. After entering the human body, LPS stimulates the innate immune system, and triggers a series of biochemical and cellular reactions that consequently lead to not only inflammation but marked toxicity in certain cases. LPS has been well documented to be a typical macrophage activator [3]. Alveolar macrophages represent the chief resident cells in the bronchoalveolar space, and respond in a rapid manner to toxic substances as well as the pathogens in lower respiratory tract functioning with alveolar epithelial cells [4]. Therefore, new mechanical insights are needed for the development of new strategies, biomarkers and improved therapies for ALI patients.

Inflammasomes are assembled by the nod-like receptors (NLRs) in response to various stimuli, including reactive oxygen species, cell stress, danger-associated molecular patterns (DAMPs), and pathogen-associated molecular patterns [5]. The family of NLRs are comprised of 14 members, NLP family pyrin domain containing 1 (NLRP1) to 14, some of which are involved in the assembly of the inflammasome, including NLRP1, NLRP3, NLRC4, NLRP6, NLRP7 and NLRP12, while NLRP3 has been found to promote the maturation of interleukin-1β (IL-1β) [6]. In addition, NLRP3 inflammasome has been highlighted in literature as being of particular significance in relation to the interaction between the innate and adaptive immunity systems [7]. More importantly, NLRP3 inflammasome has been highlighted as a promising therapeutic candidate for management of ALI [8]. It has been revealed that the exosomal miR-17 is associated with acute liver failure by targeting TXNIP or NLRP3, thus reducing inflammasome activation in hepatic macrophages [9]. A previous report revealed that microRNAs (miRs) modulate various biological functions in human cells, and mesenchymal stem cells (MSCs)-derived exosomes expressing miR-124-3p suppresses the P2X7 expression, thereby improving oxidative stress injury and inflammatory response in traumatic ALI [10]. Moreover, long non-coding RNAs (lncRNAs) have been shown to exhibit extensive regulatory effects on gene expression, including on epigenetic, transcriptional, and post-transcriptional levels [11]. Moreover, changes of lncRNAs and messenger RNAs in lung tissues of mice with LPS-induced ALI have been previously reported, whereby the lncRNA interference alleviated inflammation [12]. Furthermore, the maternally expressed gene 3 (MEG3), as an endogenous sponge, can inhibit the function of miR-223 by increasing NLRP3 expression, thus enhanced the pyrolysis of endothelial cells in atherosclerosis, an inflammatory disease associated with endothelial dysfunction [13]. Hence, based on the aforementioned exploration of literature, we subsequently hypothesized that the lncRNA MEG3/miR-7b/NLRP3 axis may play a vital role in LPS-induced ALI.

## RESULTS

### Increased expression of NLRP3 in LPS-induced *in vivo* and *in vitro* ALI models

**Figure 1:**
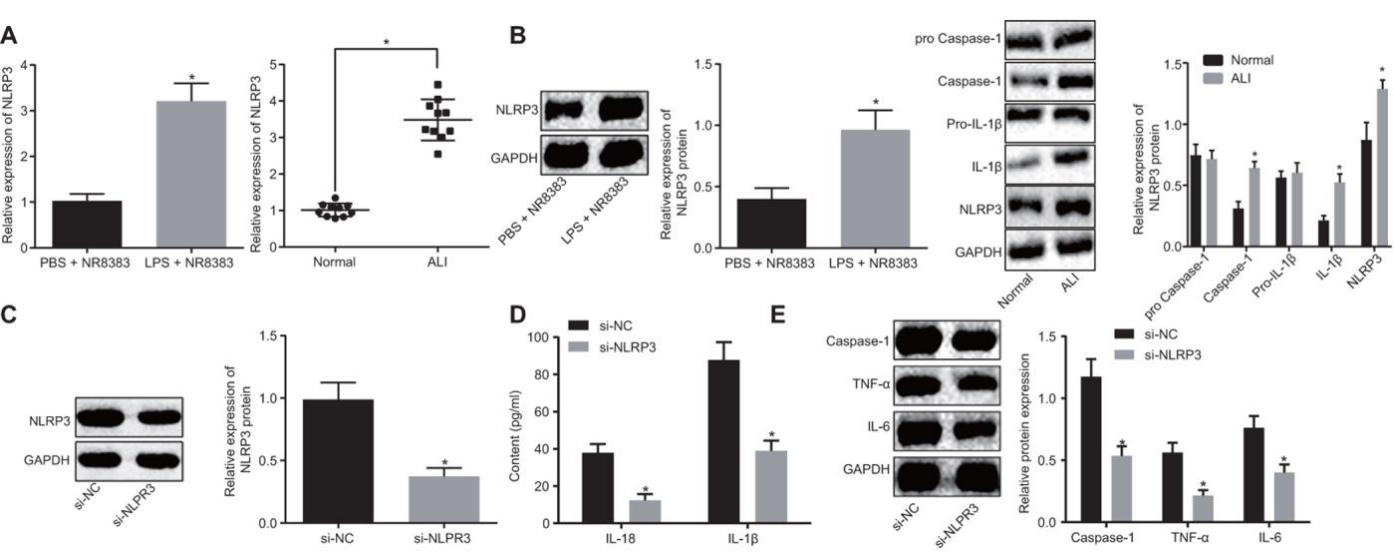
**NLRP3 was highly expressed in LPS-induced NR8383 cell and ALI mouse models.** NLRP3 expression in NR8383 cells and mice tissues with or without LPS induction assessed by RT-qPCR (N = 10) (**A**). Levels of NLRP3, pro-caspase-1/caspase-1 and pro-IL-1β/IL-1β normalized to GAPDH in alveolar macrophages (NR8383) of normal mice and mice with ALI evaluated by Western blot analysis (N = 10) (**B**). Interference efficiency of NLRP3 normalized to GAPDH evaluated by Western blot analysis (N = 10) (**C**). Levels of cytokines IL-18 and IL-1β detected by ELISA (**D**). Levels of caspase-1, TNF-α and IL-6 protein normalized to GAPDH in NR8383 cells by Western blot analysis (**E**). * *p* < 0.05 *vs*. PBS + NR8383, normal or si-NC. Measurement data were expressed as mean ± standard deviation. Comparison between two groups was conducted using unpaired *t*-test. The experiments were repeated three times independently.

LPS was initially employed to induce alveolar macrophages in NR8383 cell models and ALI mouse models. The expression of NLRP3 in non-LPS-induced and LPS-induced NR8383 cells and lung tissues of mice was detected by RT-qPCR and Western blot analysis. The results obtained (Figure 1A, 1B) illustrated that the NLRP3 expression in the LPS-induced NR8383 cells and lung tissues of the mice increased significantly (*p* < 0.05). Furthermore, Western blot analysis results revealed significantly upregulated pro-caspase-1/caspase-1 and pro-IL-1β/IL-1β in lung tissues of LPS-induced mice, which was indicative of successful ALI model establishment, accompanied by activation of NLRP3 inflammasome along with promoted maturation and release of cytokines. The NLRP3 expression was interfered in LPS-induced NR8383 cells and interference effects of si-NLRP3 was lower when compared to si-NC (Figure 1C) (*p* < 0.05).

ELISA (Figure 1D) results revealed lower IL-18 and IL-1β levels in the cells treated with si-NLRP3 in comparison to the cells treated with si-NC (*p* < 0.05). Western blot analysis (Figure 1E) indicated reduced caspase-1, TNF-α and IL-6 levels in NLRP3 silenced cells (*p* < 0.05). Altogether, our results revealed that NLRP3 was highly expressed in LPS-induced alveolar macrophages (NR8383), and the acute inflammatory response was improved after silencing NLRP3.

### miR-7b inhibits LPS-induced acute inflammation of alveolar macrophages (NR8383) in mice model of ALI

**Figure 2:**
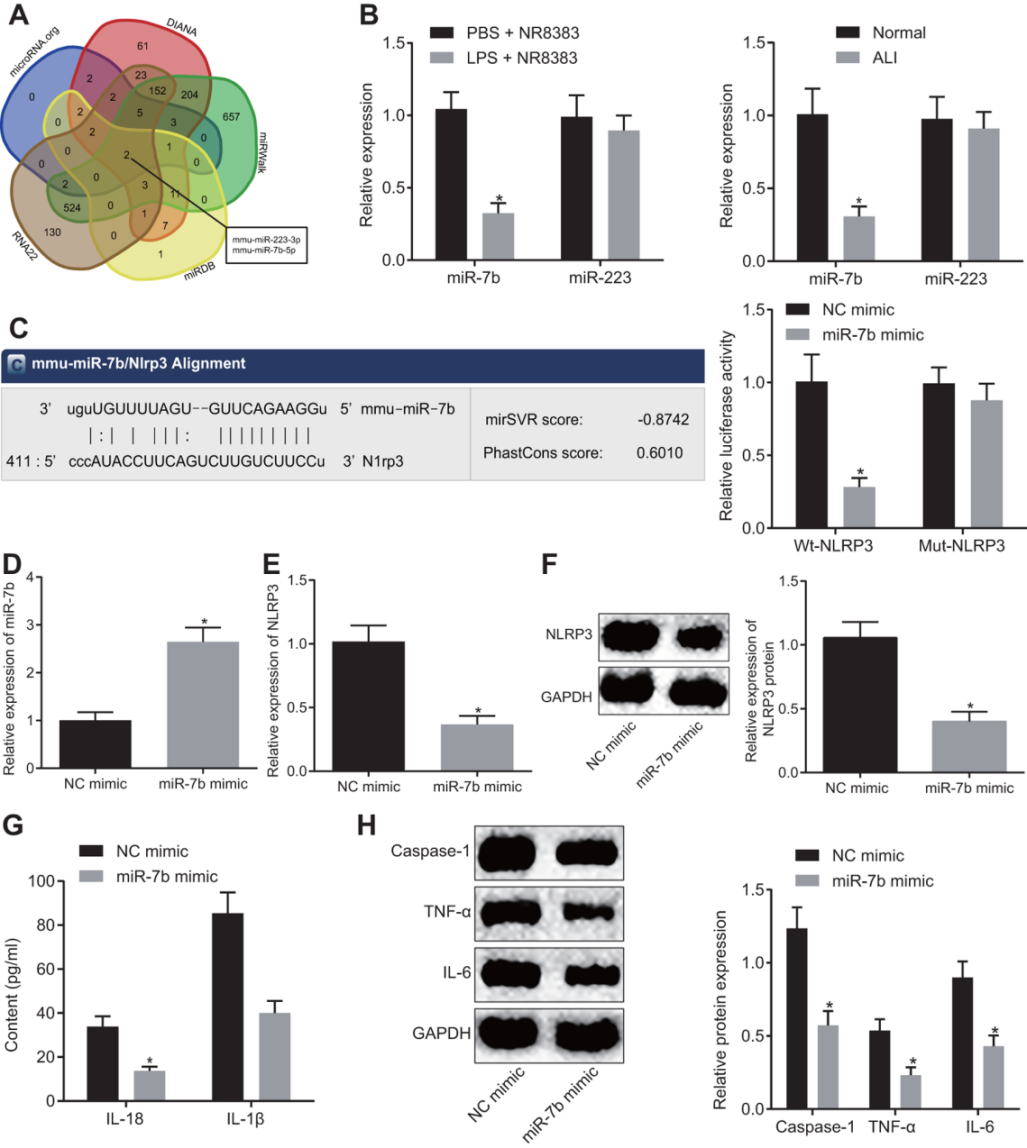
**The LPS-induced acute inflammation of alveolar macrophages (NR8383) in mice was reduced after miR-7b overexpression.** The predicted miRNA targeting NLRP3 (2 intersecting miRNAs) based on DIANA, RNA22, microDB, microWalk, and microRNA. org (**A**). miR-7b and miR-223 expression in NR8383 cells and lung tissues of mice with or without LPS induction detected by RT-qPCR (N = 10) (**B**). Dual luciferase reporter gene assay verified the targeting relationship between miR-7b and NLRP3 (**C**). RT-qPCR for efficiency of miR-7b overexpression (**D**). Expression of NLRP3 after overexpression of miR-7b detected by RT-qPCR and Western blot analysis (normalized to GAPDH) (**E**–**F**). The levels of cytokines IL-18 and IL-1β detected by ELISA (**G**). The protein expression of caspase-1, TNF-α and IL-6 expression normalized to GAPDH detected by Western blot analysis (**H**). * *p* < 0.05 *vs*. PBS + NR8383, normal or NC mimic. Measurement data were expressed as mean ± standard deviation. Comparison between two groups was conducted using unpaired *t*-test. The experiments were repeated three times independently.

Prediction of the targeted miRNAs of NLRP3 was conducted based on the results obtained from DIANA, RNA22, microDB, microWalk and microRNA.org, where a total of 481, 846, 30, 1564 and 21 miRNAs were identified, respectively. Venn diagrams were generated following analysis of the miRNAs. As depicted in Figure 2A, there were two intersecting miRNAs: mmu-miR-223-3p and mmu-miR-7b-5p. Reports have previously revealed that in mitochondrial DAMP-induced ALI, miR-223 can target NLRP3 [14], while miR-7b can inhibit cardiomyocyte apoptosis [15, 16]. This being said, the role of miR-7b in ALI remains unclear. The expression of miR-7b and miR-223 in NR8383 cells and lung tissues of mice with or without LPS induction were detected by RT-qPCR. The results obtained revealed low expression of miR-7b in the NR8383 cells and lung tissues of the LPS-induced mice (*p* < 0.05), with no significant difference detected in regard to miR-223, suggesting that miR-7b might be involved in regulating NR8383 (Figure 2B).

Dual luciferase reporter gene assay was subsequently performed, the results of which indicated that NR8383 cells introduced with miR-7b and wt-NLRP3 had lower fluorescence intensity than that treated with NC mimic and wt-NLRP3, highlighting the targeting relationship between miR-7b and NLRP3 (*p* < 0.05) (Figure 2C). RT-qPCR revealed that miR-7b overexpression in LPS-induced NR8383 cells led to an increased transfection efficacy (*p* < 0.05) (Figure 2D).

The RT-qPCR and Western blot analysis (Figure 2E, 2F) demonstrated that miR-7b overexpression led to a reduction in the expression of NLRP3 (*p* < 0.05). ELISA (Figure 2G) revealed that overexpressed miR-7b triggered a decrease in the expression of IL-18 and IL-1β (*p* < 0.05). Western blot analysis revealed that the expression of caspase-1, TNF-α and IL-6 was downregulated after miR-7b overexpression (*p* < 0.05) (Figure 2H). Thus, the low expression of miR-7b in LPS-induced alveolar macrophages (NR8383) of ALI mice was observed, while the acute inflammatory response was improved following miR-7b overexpression.

### LncRNA MEG3 sponges miR-7b to regulate NLRP3 expression

**Figure 3:**
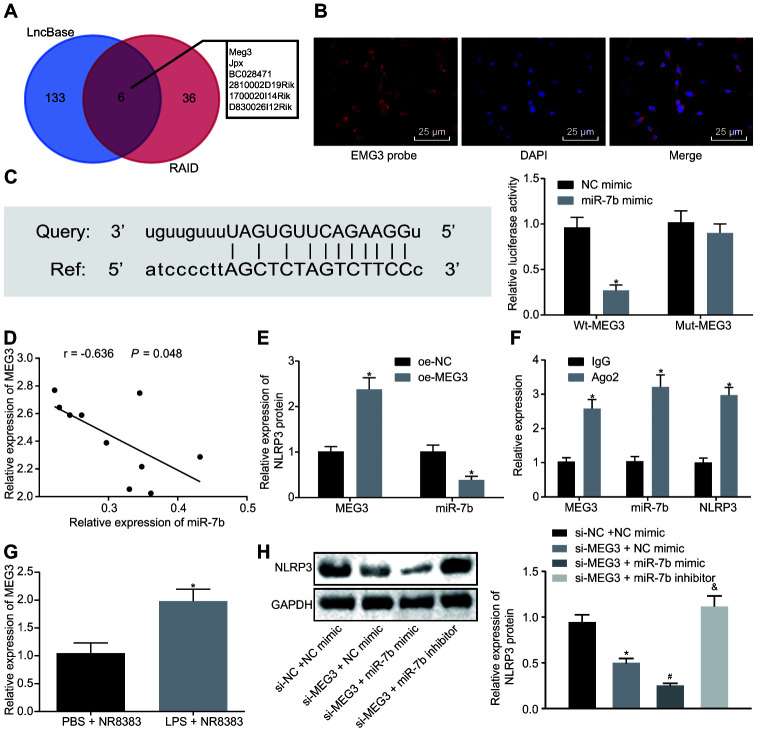
**LncRNA MEG3 binds to miR-7b and competes with NLRP3.** RAID and LncBase predicted lncRNAs that might bind to miR-7b (**A**). RNA-FISH for lncRNA MEG3 expression in NR8383 cells (× 200) (**B**). Dual luciferase reporter gene assay verified the targeting relationship between miR-7b and NLRP3 (**C**). RT-qPCR for targeting relation of miR-7b and lncRNA MEG3, * *p* < 0.05 *vs*. NC mimic (**C**). Correlation analysis of miR-7b and lncRNA MEG3 expression (**D**). The expression of miR-7b after lncRNA MEG3 overexpression by RT-qPCR, * *p* < 0.05 *vs*. oe-NC (**E**). RIP assay for the binding of miR-7b with lncRNA MEG3 or NLRP3 respectively, * *p* < 0.05 *vs*. IgG (**F**). The expression of lncRNA MEG3 in LPS-induced cells was detected by RT-qPCR, * *p* < 0.05 *vs*. PBS + NR8383 (**G**). The NLRP3 expression normalized to GAPDH detected by Western blot analysis, * *p* < 0.05 *vs*. si-NC + NC mimic, # *p* < 0.05 *vs*. si-MEG3 + NC mimic, & *p* < 0.05 *vs*. si-MEG3 + miR-7b mimic (**H**). Measurement data were expressed as mean ± standard deviation. Comparison between two groups was conducted using unpaired *t*-test. Data among multiple groups were tested using ANOVA, followed by Tukey’s post hoc test. The experiments were repeated three times independently.

The potential targets of lncRNAs on miR-7b were predicted using RAID and LncBase. The results illustrated that there were six intersecting lncRNAs, namely MEG3, Jpx, BC028471, 2810002D19Rik, 1700020I14Rik, and D830026I12Rik (Figure 3A). Silencing MEG3 has been previously reported to inhibit ox-LDL-induced macrophage inflammation and apoptosis [17], while the overexpression of MEG3-4 has been shown to aggravate inflammation and lung injury [18]. Hence, we set out to investigate the potential role of lncRNA MEG3 in ALI and speculated that lncRNA MEG3 may adsorb miR-7b to regulate NLRP3. The subcellular localization of NLRP3 in mouse alveolar macrophages was identified by FISH (Figure 3B), which indicated that NLRP3 was expressed in both the nucleus and the cytoplasm.

The bioinformatics website predicted that lncRNA MEG3 had a targeting relationship with miR-7b, and dual luciferase reporter gene assay (Figure 3C) which further confirmed that the co-transfection of miR-7b and wt-MEG3 resulted in decreased fluorescence intensity (*p* < 0.05). These results verified a targeting relationship between miR-7b and lncRNA MEG3.

Correlation analysis of miR-7b and lncRNA MEG3 (Figure 3D, 3E) showed that the expression of miR-7b was negatively correlated with lncRNA MEG3 expression, while the overexpression of lncRNA MEG3 led to a decrease in the expression of miR-7b. In concert with previous results, RIP assay was employed to detect the binding of miR-7b to lncRNA MEG3 or NLRP3, respectively (Figure 3F). The results obtained suggested that anti-Ago2 elevated the binding of miR-7b to lncRNA MEG3 or NLRP3 (*p* < 0.05). Upregulation of lncRNA MEG3 was identified in LPS-induced NR8383 cells (Figure 3G).

Western blot analysis (Figure 3H) further confirmed that the silenced lncRNA MEG3 reduced the NLRP3 level, while the co-treatment of NLRP3 and overexpression of miR-7b brought about a more distinct trend (*p* < 0.05). Compared with the presence of silenced lncRNA MEG3 or silenced lncRNA MEG3 + miR-7b mimic, NLRP3 expression was restored in the presence of silenced lncRNA MEG3 + miR-7b inhibitor. Therefore, lncRNA MEG3 acts as the internal competitive RNA of miR-7b. When lncRNA MEG3 was downregulated, miR-7b bound to NLRP3 more and inhibited the expression of NLRP3.

### LncRNA MEG3/miR-7b regulates LPS-induced ALI *in vitro* through NLRP3

**Figure 4:**
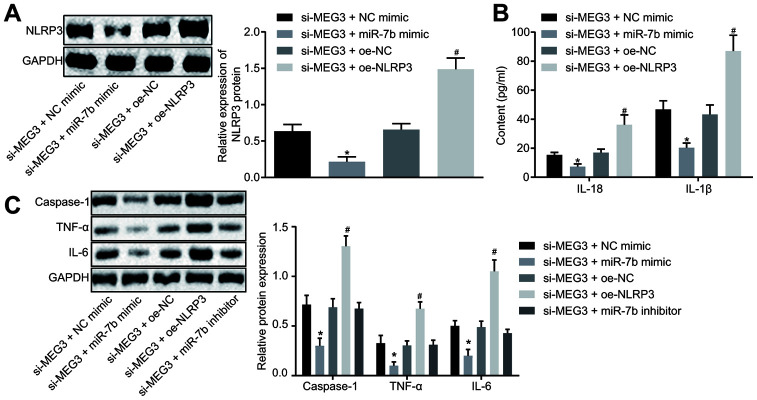
**LncRNA MEG3 sponged miR-7b through NLRP3 to modulate the LPS-induced ALI cells *in vitro*.** The expression of NLRP3 normalized to GAPDH assessed by Western blot analysis (**A**). The levels of cytokines IL-18 and IL-1β detected by ELISA (**B**). The expression of caspase-1, TNF-α and IL-6 normalized to GAPDH detected by Western blot analysis (**C**). * *p* < 0.05 *vs*. si-MEG3 + NC mimic; # *p* < 0.05 *vs*. si-MEG3 + oe-NC. Measurement data were expressed as mean ± standard deviation. Data among multiple groups were tested using ANOVA, followed by Tukey’s post hoc test. The experiments were repeated three times independently.

LPS-induced NR8383 cells were treated with si-MEG3, miR-7b mimic, or oe-NLRP3 together or separately in order to further elucidate their interactions in ALI. Western blot analysis (Figure 4A) revealed that the NR8383 cells treated with both si-MEG3 and miR-7b mimic exhibited downregulated NLRP3 expression, while NR8383 cells treated with both si-MEG3 and oe-NLRP3 displayed an opposite trend (*p* < 0.05).

ELISA was conducted to determine the expression levels of IL-18 and IL-1β, which revealed that the treatment of both si-MEG3 and miR-7b mimic reduced the expression of IL-18 and IL-1β, while treatment with both si-MEG3 and oe-NLRP3 elevated the expression of IL-18 and IL-1β in NR8383 cells (*p* < 0.05) (Figure 4B).

Western blot analysis (Figure 4C) illustrated that NR8383 cells administered with si-MEG3 and miR-7b mimic had decreased levels of caspase-1, TNF-α and IL-6 (*p* < 0.05), while NR8383 cells administered with si-MEG3 and oe-NLRP3 exhibited contrasting results (*p* < 0.05). Moreover, delivery of si-MEG3 + miR-7b inhibitor significantly restored the expression of NLRP3, caspase-1, TNF-α and IL-6 affected by si-MEG3 or si-MEG3 + miR-7b mimic. These results indicated that lncRNA MEG3 could sponge miR-7b and upregulate the expression of NLRP3. After interfering with lncRNA MEG3, miR-7b inhibited NLRP3 expression to improve LPS-induced ALI *in vitro*.

### LncRNA MEG3/miR-7b regulates LPS-induced ALI *in vivo* *via* NLRP3

**Figure 5:**
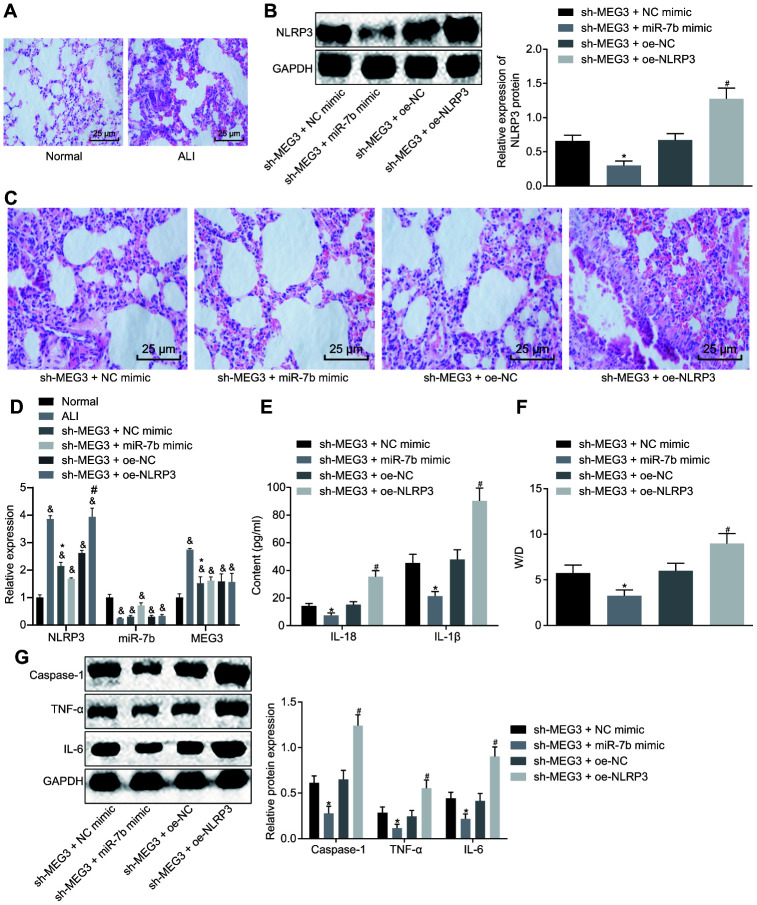
**LncRNA MEG3 sponged miR-7b to upregulate NLRP3 to modulate LPS-induced ALI in mice.** The pathological changes of lung tissues were observed after the HE staining (× 200) (**A**, **C**). The expression of NLRP3 normalized to GAPDH assessed by Western blot analysis (**B**). The expression of NLRP3, miR-7b and MEG3 in lung tissues of mice following different treatment protocols determined by RT-qPCR (**D**). The levels of cytokines IL-18 and IL-1β detected by ELISA (**E**). The pulmonary edema determination of dry weight and wet weight (**F**). The expression of caspase-1, TNF-α and IL-6 normalized to GAPDH detected by Western blot analysis (**G**). & *p* < 0.05 *vs*. normal; * *p* < 0.05 *vs*. si-MEG3 + NC mimic; # *p* < 0.05 *vs*. si-MEG3 + oe-NC. Measurement data were expressed as mean ± standard deviation. Data among multiple groups were tested using ANOVA, followed by Tukey’s post hoc test. The experiments were repeated three times independently. N = 10.

Following successful ALI mouse model establishment, the pathological results of the lung tissues were analyzed after HE staining (Figure 5A, 5C). No evidence of thickening or lymphocyte infiltration was identified in the normal mice. On the other hand, distinct pathological changes like alveolar wall thickening, alveolar collapse, a large number of red blood cells and inflammatory cells infiltration was observed in the lung tissues of ALI modeled mice, all of which was indicative of successful ALI model establishment. In mice treated with short hairpin (sh)-MEG3 and miR-7b mimic, alleviated pathological signs as well as a reduction in inflammatory cell infiltration were identified in the lung tissues. The mice treated with sh-MEG3 + NC mimic or sh-MEG3 + oe-NC exhibited only slightly alleviated pathological changes. Mice with sh-MEG3 + oe-NLRP3 treatment displayed the most distinct lung tissue lesions, alveolar wall thickening, alveolar collapse, and a large number of red blood cells and inflammatory cell infiltration.

Western blot analysis of NLRP3 interference efficiency (Figure 5B) showed an increase in the expression of NLRP3 in the mice treated with sh-MEG3 + oe-NLRP3 (*p* < 0.05). Moreover, RT-qPCR results revealed significantly upregulated NLRP3 and downregulated miR-7b in the lung tissues of mice with ALI when compared to the normal mice, while delivery of sh-MEG3 + miR-7b mimic induced higher miR-7b expression and lower NLRP3 expression (Figure 5D). ELISA (Figure 5E) results revealed that the levels of IL-18 and IL-1β in mice treated with both sh-MEG3 and miR-7b mimic were reduced, while mice treated with sh-MEG3 and oe-NLRP3 showed the opposite trends.

The pulmonary edema determination of dry weight and wet weight are depicted in Figure 5F. When compared to the mice treated with sh-MEG3 and NC mimic, the W/D in mice with treatment of sh-MEG3 and miR-7b mimic was significantly lower (*p* < 0.05), while mice treated with sh-MEG3 and oe-NLRP3 exhibited a higher W/D than the mice treated with sh-MEG3 and oe-NC (*p* < 0.05).

Western blot analysis (Figure 5G) demonstrated that the levels of caspase-1, TNF-α and IL-6 were significantly downregulated in mice introduced with sh-MEG3 and miR-7b mimic when compared to mice administered with sh-MEG3 and NC mimic (*p* < 0.05), while the mice treated with sh-MEG3 and oe-NLRP3 exhibited opposite results when compared with the mice treated with sh-MEG3 and oe-NC (*p* < 0.05). In summary, lncRNA MEG3 sponged miR-7b to upregulate the NLRP3 expression. After silencing of lncRNA MEG3, miR-7b suppressed NLRP3 to improve LPS-induced ALI *in vivo*.

## DISCUSSION

ALI is a serious clinical condition marked by acute respiratory distress and deficient surfactant proteins, often leading to increased chronic pulmonary fibrosis [19]. ALI has been well documented to be characterized by the release of inflammatory mediators following epithelial cell injury, advancing initial influx of neutrophils and macrophages into the injury site, which further elevates the production of cytokines and regulation of extracellular matrix, including fibronectin, hyaluronan, elastin, and collagen [20]. The present study reported that lncRNA MEG3 could sponge miR-7b, and lncRNA MEG3 silencing or overexpression of miR-7b may led to the downregulation of NLRP3, thus reducing the expression levels of IL-18, IL-1β, caspase-1, TNF-α and IL-6, ultimately resulting in the improved LPS-induced ALI.

The NLRP3 inflammasomes were activated by the cellular stresses *via* two-component pathway, the first of which was interaction with Toll-like receptor 4-ligand, followed by a second signal, such as ATP-dependent P2X purine receptor 7 receptor activation. Although NLRP3 inflammasome activation has been emphasized as an important component in the host defense mechanism against invasive bacteria and pathogens, the over activation of inflammasomes may induce inflammation-related tissue damage in chronic disease environment [21]. Our results revealed that NLRP3 was highly expressed in LPS-induced NR8383 cell ALI model. NLRP3 silencing was observed to inhibit the protein levels of IL-18, IL-1β, caspase-1, TNF-α and IL-6 to reduce inflammation. LPS-induced ALI was characterized by the release of pro-inflammatory mediators, which coordinates the inflammatory responses through the interaction of LPS and immune cells. After entry into the alveolar cavity, LPS has been shown to interact with alveolar macrophages triggering the activation of cells as well as their inflammatory mediators, including IL-1β and IL-1α [2]. Likewise, upregulation of IL-1β and IL-18 was indicative of activation of caspase-1 and inflammasome following LPS induction in the liver [22]. Furthermore, the NLRP3 inflammasome has been reported as a multiprotein complex capable of mediating the maturation of IL-1β and IL-18 on the premise of the caspase-1 activation [23]. Furthermore, it was found that NLRP3 and caspase-1 expression in rats was decreased by adenovirus-Angiopoietin-1 treatment, reducing the levels of IL-1b, IL-18 and IL-33 in both serum and bronchoalveolar lavage fluid samples from phosgene-induced adenovirus-Angiopoietin-1-treated rats with ALI [24].

Accumulating evidence has demonstrated the essential role played by miRNAs in the pathogenesis of vascular inflammation and respiratory diseases, including ALI [25]. Study has revealed that in LPS-stimulated mice, the intranasal delivery of miR-144 mimic through liposome can alleviate endotoxemia-induced lung W/D ratio and inflammation in inflammatory lung injury [26]. In the present study, low expression of miR-7b was detected in LPS-induced alveolar macrophages (NR8383) of mice with ALI, while miR-7b overexpression led to a reduction in the expression of NLRP3, ultimately improving the acute inflammatory response. Recent research has revealed that miR-223 blocks the differentiation of LY6G + neutrophils derived from bone marrow and the release of cytokines in peripheral blood by targeting NLRP3 expression inhibition and the release of IL-1β, thus alleviating mitochondrial damage-associated molecular patterns-induced ALI [14]. Moreover, Dex treatment upregulates the expression of miR-381 by targeting NLRP3, resulting in alleviated lung injury and inhibited inflammatory factors expression, evidenced by diminished levels of NACHT, LRR, NLRP3 and autocleavage of caspase-1 [27]. Moreover, the reduction of the W/D of lung tissues, activity of methylenedioxyamphetamine, and levels of inflammatory factors (TNF-α, IL-6, and IL-8) by MSCs-derived exosomal miR-124-3p in mice was observed, thus resulting in relieved oxidative stress injury as well as suppressed inflammatory response in traumatic ALI [10].

A key finding of the present study revealed that lncRNA MEG3 sponges miR-7b to regulate the expression of NLRP3, thus improving ALI. LncRNAs represent a class of non-protein-coding transcripts that have been implicated in a wide variety of biological processes, including miRNA silencing, with lncRNA MEG3 expression detected in many human tissues [28]. LncRNAs represent a new class of heterologous ncRNAs, which are composed of intron/exon lncRNAs, antisense lncRNAs, overlapping lncRNAs and lincRNAs. In ncRNAs, both lncRNAs and miRNAs show strong tissue specificity and are associated with complex diseases, including the application of hepatocyte MSCs and FTY720 in LPS-induced ALI treatment [11].

**Figure 6:**
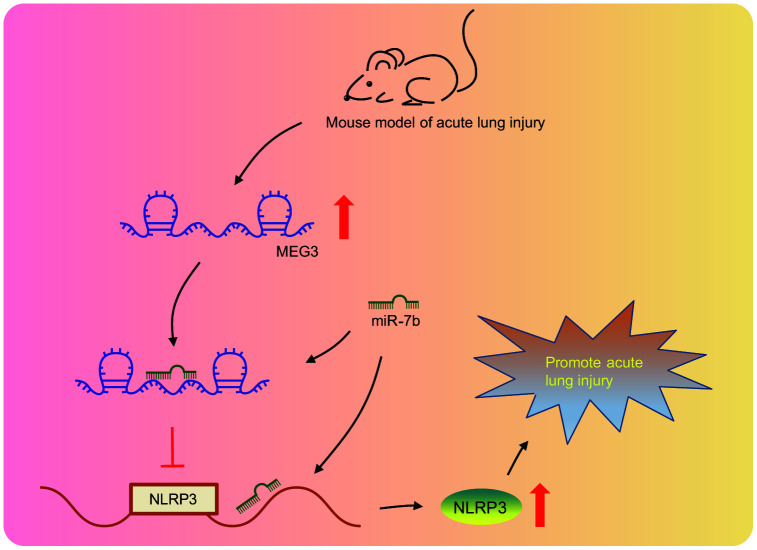
**LncRNA MEG3 was highly expressed in LPS-induced ALI mouse models.** LncRNA MEG3 sponged miR-7b to upregulate NLRP3 ultimately promoting LPS-induced ALI.

Taken together, our findings provided evidence that lncRNA MEG3 acts as the internal competitive RNA of miR-7b. When lncRNA MEG3 was downregulated, the binding of miR-7b to NLRP3 increased and the lncRNA NLRP3 expression was inhibited, thus resulting in improved LPS-induced ALI (Figure 6). Our study proposes novel biological markers for the treatment of ALI, which could potentially provide a new perspective regarding the theoretical basis of the NLRP3 mechanism. However, our study was performed based on the model of LPS-induced ALI in cells and mice, with the clinical effects of the lncRNA MEG3/miR-7b/NLRP3 axis in LPS-induced ALI treatment still largely unknown.

## MATERIALS AND METHODS

### Ethics statement

The protocol of the current study was approved by the Experimental Animal Ethics Committee of Shanghai Ninth People’s Hospital, Shanghai JiaoTong University School of Medicine. All animal experiments were conducted with extensive efforts made to minimize the pain and number of experimental animals.

### Establishment of ALI cell models *in vitro*

Mouse alveolar macrophages (NR8383, Beijing Union Bio-engineering Institute, Chinese Academy of Medical Sciences) were cultured in Ham’s S F-12K complete medium containing 1.59/L sodium bicarbonate, 15% fetal bovine serum and 2 m ML-glutamine at 37°C in 5% CO_2_. The medium was renewed every 2 - 3 d.

Cell concentration was adjusted to 4 × 10^5^ cells/mL, after which the cells (100 mL/well) were inoculated into a 96-well plate and cultured for 1 h. The cells treated with 12K culture medium were employed as the control, while the cells treated with LPS with a final concentration of 100 μg/mL for 18 h were used to establish the ALI cell model. The cell density was adjusted after which the cells were then inoculated into a 6-well plate. The cells were then transfected with lipofectamine 2000 (Invitrogen, Carlsbad, CA, USA) kit and detected after 48 h of incubation.

### Establishment of LPS-induced ALI *in vivo* animal model

A total of 70 male, 7-week-old C57 BL/6J mice of specific pathogen free grade were purchased from Beijing Solarbio Technology Co., Ltd. (Beijing, China). Ten mice were employed as the control, while 60 mice were used to establish ALI model. The ALI model was established by intratracheal drip of 7.5 mg/kg LPS into the lungs of mice [29]. Briefly, the mice were anaesthetized by intraperitoneal injection of 3% isopentobarbital solution (100 mg/kg), and placed in a supine position. A 0.5 cm longitudinal incision was made 1 - 1.5 cm away from the lower teeth along the middle of the neck in order to expose the trachea. LPS solution mixed in 300 μL sterile saline was then injected into the trachea. Next, the control mice were injected with 300 μL sterile saline. Only 52 mice (86.67%) were deemed to have been successfully established. After 6 h of animal model establishment, 50 of the successfully modeled mice (two for backup) were administered with a tail-vein injection with overexpressed (oe)-MEG3, miR-7b mimic, miR-7b inhibitor and NLRP3 interference plasmids (all from Zhonghong Boyuan Biotechnology Co., Ltd., Shanghai, China). After 48 h, tracheal intubation was performed on the mice. The alveoli were subjected to lavage with 1.2 mL phosphate buffer saline (PBS) for three times, with the lavage solution then collected. The mice were then euthanized to determine the degree of lung injury.

### RNA-fluorescence in situ hybridization (FISH)

The subcellular localization of lncRNA MEG3 in the mouse alveolar macrophages was detected by FISH using Ribo^TM^ lncRNA FISH Probe Mix (Red) (C10920, Guangzhou RiboBio Co., Ltd., Guangzhou, Guangdong, China). Briefly, the cells (6 × 10^4^ cells/well) were inoculated into a 24-well plate when the cell confluence reached approximately 60% - 70%. The cells in each well were then fixed with 1 mL of 4% polyformaldehyde for 10 min, added with 1 mL of pre-cooled PBS containing 0.5% Triton X-100 at 4°C for 5 min, and blocked with 200 μL pre-hybridization solution at 37°C for 30 min, followed by overnight incubation with probe hybridization solution containing probe (anti-MEG3 nucleotide probe, Wuhan GeneCreate Biological Engineering Co., Ltd., Wuhan, Hubei, China) at 37°C. The cells were subsequently washed with 42°C wash liquid I-III, stained with 4’6-diamidino-2-phenylindole (1 : 800) for 10 min, sealed with nail polish, and finally analyzed and photographed under a fluorescence microscope (Olympus, Tokyo, Japan) in 5 randomly selected visual fields.

### Dual luciferase reporter gene assay

The wild type (wt) and mutant type (mut) reporter plasmids of lncRNA MEG3 and NLRP3 were designed by the Shanghai GenePharma Co., Ltd. (Shanghai, China). Negative control (NC) mimic and miR-7b mimic were co-transfected with wt-NLRP3, mut-NLRP3, wt-MEG3 and mut-MEG3 into 293T cells respectively. After 48 h, the cells were collected and the change of luciferase activity was detected using a Genecopoeia’s dual luciferase detection kit (D0010, purchased from Beijing Solarbio Technology Co., Ltd., Beijing, China). The fluorescence intensity was measured using a GLomax 20/20 Luminometer (E5311, Shaanxi Zhongmei Biotechnology Co., Ltd., Shaanxi, China).

### Bioinformatics analysis

The NLRP3 inflammasome has been previously reported to play a role in autoimmune, infectious and metabolic diseases, including ALI [8]. Studies have demonstrated that inhibiting NLRP3 can alleviate ALI [30, 31]. In order to further explore the possible molecular mechanism by which NLRP3 contributes to ALI, the possible miRNA regulators of NLRP3 were predicted based on data from DIANA (http://diana.imis.athena-innovation.gr/DianaTools/index.php?r=microT_CDS/index), RNA22 (http://ophid.utoronto.ca/mirDIP/), miRDB(http://www.mirdb.org/), miRWalk (http://mirwalk.umm.uni-heidelberg.de/), and microRNA.org. Venn diagrams (http://bioinformatics.psb.ugent.be/webtools/Venn/) were subsequently plotted and used to compare the predicted results of miRNAs for the miRNA screening. The predicted bindings of lncRNA to miRNAs were also compared and subsequently screened using both RAID (http://www.rna-society.org/raid/index.html) and LncBase (http://carolina.imis.athena-innovation.gr/diana_tools/web/index.php?R=lncev2%2Findex-predicted).

### RNA immunoprecipitation (RIP) assay

The cells were added with RIP lysis buffer (N653-100 mL, Shanghai Haoran Bio Technologies Co., Ltd., Shanghai, China) and lysed on ice for 5 min. The lysed cells were initially added with 50 μL magnetic beads, after which 0.5 mL RIP wash buffer (EHJ-BVIS08102, Xiamen Huijia Biotechnology Co., Ltd., Xiamen, Fujian, China) was added and placed on the magnetic separator for bead aggregation. The beads were then incubated with 5 μg Argonaute 2 (Ago2) antibody (P10502500, Otwo Biotech Inc., Shenzhen, Guangdong, China) for 30 min with normal mouse immunoglobulin G (IgG) regarded as the NC. After the supernatant had been discarded, the beads were washed with 0.5 mL RIP wash buffer. The beads-antibody complexes were then added with 900 μL RIP immunoprecipitation buffer (P10403138, Otwo Biotech Inc., Shenzhen, Guangdong, China) with the complexes incubated at 4°C overnight, washed with 0.5 mL RIP wash buffer, and permitted to react with 150 μL proteinase K buffer at 55°C for 30 min in order to purify the RNA for reverse transcription quantitative polymerase chain reaction (RT-qPCR).

### Enzyme linked immunosorbent assay (ELISA)

The cell concentration was adjusted to 4 × 10^5^ cells/mL, and incubated in a 24-well plate for 1 h (500 μL/well). LPS was added to each well and co-cultured for 24 h. The cell culture medium was then collected, and centrifuged followed by collection of the supernatant. After tracheal intubation, the alveoli of mice were subjected to lavage with 1.2 mL PBS for three times. The lavage fluid was collected in a cryogenic centrifuge at 4°C for 10 min. The supernatant was then preserved at -20°C. The levels of cytokines IL-18 and IL-1β were then detected and recorded.

### Measurement of pulmonary edema

The left lungs of mice without alveolar lavage were removed after thoracotomy and weighed. After baking at 80°C for 48 h, the lungs were weighed again. The ratio of wet weight to dry weight (W/D) and lung water content were subsequently calculated and considered to be reflective of the degree of pulmonary edema. W/D = [wet/dry weight of lung] × 100%.

### Pathological observation of lung tissues

The left lungs of the mice without alveolar lavage were fixed by formalin, sliced at 5 μm, and baked at 60°C for 1 h before dewaxed by xylene. After hydration, the routine hematoxylin-eosin (HE) (Beijing Solarbio Technology Co., Ltd., Beijing, China) staining was performed, followed by gradient alcohol dehydration, xylene clearing, and neutral gum sealing. The pathological changes were then observed under an optical microscope (XP-330, Bingyu Optical Instruments Co., Ltd., Shanghai, China).

### RT-qPCR

Total RNA was extracted using the TRIzol method followed by reverse transcription into complementary DNA (cDNA) using a PrimeScript RT kit (RR036A, Takara, Shiga, Japan). The primers used (Table 1) were synthesized by (Takara, Shiga, Japan). RT-qPCR was performed using SYBR^®^ Premix Ex Taq^TM^ II kit (RR820A, TaKaRa, Shiga, Japan) on ABI7500 quantitative instrument (7500, ABI, USA). U6 was considered as the internal control for the miRNAs while glyceraldehyde-3-phosphate dehydrogenase (GAPDH) for others. The relative expression was calculated using the 2^-ΔΔCt^ method.

**Table 1:** Primer sequences for RT-qPCR.

	**Primer sequences (5'-3')**
MEG3	F: TGGGGATGGGTCTCTAGGTG
	R: CCACTGACCCACAGTAACCC
miR-7b	F: ACGTGAGCCAGTGCTATGTG
	R: GAGTCCACGCTATGAGGCTG
miR-223	F: TCACGCTCCGTGTATTTGACA
	R: CATGAGCCACACTTGGGGTAT
NLRP3	F: TCTGCACCCGGACTGTAAAC
	R: CATTGTTGCCCAGGTTCAGC
U6	F: TCGCACAGACTTGTGGGAGAA
	R: CGCACATTAAGCCTCTATAGTTACTAGG
GAPDH	F: TTAGCACCCCTGGCCAAGG
	R: CTTACTCCTTGGAGGCCATG

Note: MEG3, maternally expressed gene 3; miR-7b, microRNA-7b; miR-223, microRNA-223; NLRP3, NLR pyrin domain containing 3; GAPDH, glyceraldehyde-3-phosphate dehydrogenase; RT-qPCR, reverse transcription quantitative polymerase chain reaction; F, forward; R, reverse.

### Western blot analysis

Total proteins were extracted from tissues or cells by radio immunoprecipitation assay lysis buffer containing phenylmethyl-sulfonyl fluoride, with the protein concentration measured using bicinchoninic acid kits. The protein was then subjected to sodium dodecyl sulfate-polyacrylamide gel electrophoresis and transferred onto polyvinylidene fluoride membranes. The membranes were then blocked using 5% skimmed milk for 1 h, and incubated at 4°C overnight with primary antibodies of rabbit anti-mouse NLRP3 (ab214185, 1 : 100), caspase-1 (ab1872, 1 : 1000), tumor necrosis factor-α (TNF-α) (ab6671, 1 : 1000), IL-6 (ab208113, 1 : 1000), and GAPDH (ab9485, 1 : 2500). The membranes were subsequently incubated with horseradish peroxidase (HRP)-labeled secondary antibody goat-anti rabbit IgG H&L (HRP) (ab97051, 1 : 2000) for 1 h. The antibodies used were all purchased from Abcam Inc. (Cambridge, UK). The membranes were then developed using enhanced chemiluminescence after which they were imaged using Bio-Rad imaging analysis system (Bio-Rad, Hercules, CA, USA) and finally analyzed using the Quantity One v4.6.2 software.

### Statistical analysis

The data were processed using the SPSS 21.0 (IBM Corp., Armonk, NY, USA). Measurement data were expressed as mean ± standard deviation. The unpaired-design data conforming to normal distribution and homogeneity of variance between two groups were analyzed using an unpaired *t*-test. Data among multiple groups were assessed using one-way analysis of variance (ANOVA), followed by Tukey’s post hoc test. The correlation between indicators was analyzed by Pearson Correlation analysis. A value of *p* < 0.05 was considered to be indicative of statistical significance.
